# CircRNA: functions and properties of a novel potential biomarker for cancer

**DOI:** 10.1186/s12943-017-0663-2

**Published:** 2017-05-23

**Authors:** Shujuan Meng, Hecheng Zhou, Ziyang Feng, Zihao Xu, Ying Tang, Peiyao Li, Minghua Wu

**Affiliations:** 1 Hunan Provincial Tumor Hospital and the Affiliated Tumor Hospital of Xiangya Medical School, Central South University, Changsha, 410013 Hunan China; 2 The Key Laboratory of Carcinogenesis of the Chinese Ministry of Health, The Key Laboratory of Carcinogenesis and Cancer Invasion of the Chinese Ministry of Education, Cancer Research Institute, Central South University, Changsha, Hunan 410008 China

**Keywords:** circRNA, Non-coding RNA, microRNA sponge, Tumour, Biomarker

## Abstract

Circular RNAs, a novel class of endogenous noncoding RNAs, are characterized by their covalently closed loop structures without a 5′ cap or a 3′ Poly A tail. Although the mechanisms of circular RNAs’ generation and function are not fully clear, recent research has shown that circular RNAs may function as potential molecular markers for disease diagnosis and treatment and play an important role in the initiation and progression of human diseases, especially in tumours. This review summarizes some information about categories, biogenesis, functions at the molecular level, properties of circular RNAs and the possibility of circular RNAs as biomarkers in cancers.

## Background

Circular RNAs (circRNAs) were first found in RNA (ribonucleic acid) viruses as a viroid as early as 1976 [1] and were later found to be an endogenous RNA splicing product in eukaryotes in 1979 as well [2]. They were thought to be a result of splicing errors for several decades after the 1970s [1]. In the twenty-first century, with the development of RNA sequencing (RNA-seq) technologies and bioinformatics, the abundance and diversity of circRNAs was identified, and the dynamic expression patterns of circRNAs were revealed in various developmental stages and physiological conditions. More circRNA functions were also found, such as acting as scaffolds in the assembly of protein complexes [3], sequestering proteins from their native subcellular localization [4], modulating the expression of parental genes [5–8], regulating alternative splicing [9] and RNA–protein interactions [10], and functioning as microRNA (miRNA) sponges [8, 11–15]. Additionally, some researchers have found that circRNAs are abundant in eukaryotic cells, especially in mammalian brains [16], and some circRNAs are reported to be associated with human neurodegenerative diseases [17]. Recent studies have shown that circRNAs participate in the initiation and progression of tumours [18, 19].

Unlike linear RNAs, circular RNAs have a special circular covalently bonded structure, which give them a higher tolerance to exonucleases. Due to their conservation, abundance and tissue specificity, circRNAs may play roles as special molecular markers in some diseases, including tumours [20]. Here, we review the formation and function of circRNAs and discuss the possibility of circRNAs as biomarkers in cancer.

### Categories and biogenesis of CircRNAs

CircRNAs are divided into four categories: exonic circRNAs (ecircRNA), circular RNAs from introns, exon-intron circRNAs (EIciRNA) and intergenic circRNAs [21].

**Fig. 1:**
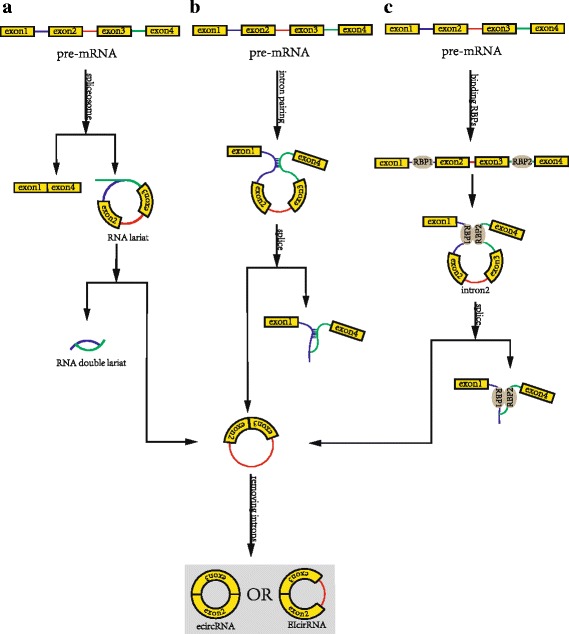
Biosynthesis of ecircRNA and EIciRNA. **a** Exon-skipping or lariat-driven circularisation. First, a pre-mRNA is spliced, causing the 3′-hydroxyl of the upstream exon to covalently bond to the 5′-phosphate of the downstream exon. At the same time, the sequence between the exons becomes an RNA lariat containing several exons and introns. Second, in the RNA lariat, the 2′-hydroxyl of the 5′-intron reacts with the 5′-phosphate of the 3′-intron, followed by the 3′-hydroxyl of the 3′-exon reacting with the 5′-phosphate of the 5′-exon. As a result, an RNA double lariat and a circular RNA are produced. Finally, some introns of the circular RNA are removed, producing an ecircRNA or EIcirRNA. **b**. Direct back-splicing or intron-pairing-driven circularisation. First, the upstream intron pairs with the downstream intron. Second, the 2′-hydroxyl of the upstream intron reacts with the 5′-phosphate of the downstream intron, followed by the 3′-hydroxyl of the 3′-exon reacting with the 5′-phosphate of the 5′-exon. Thus, a circular RNA is produced. Finally, some introns of the circular RNA are removed, producing an ecircRNA or EIcirRNA. **c**. RNA-binding-protein-driven circularisation. First, RNA binding proteins (RBPs) bind the upstream and downstream introns. Second, the RBPs are attracted to each other, and form a bridge between the introns. Third, the 2′-hydroxyl of the upstream intron reacts with the 5′-phosphate of the downstream intron, followed by the 3′-hydroxyl of the 3′-exon reacting with the 5′-phosphate of the 5′-exon. Thus, a circular RNA is produced. Finally, some introns of the circular RNA are removed, producing an ecircRNA or EIcirRNA
1.2 Circular RNAs from introns include circular intronic RNAs (ciRNAs), excised group I introns, excised group II introns, intron lariats, and excised tRNA introns [23]. The synthesis pathway of ciRNAs, excised group I introns and excised group II introns are shown in (Fig. 2).

1.1. Most ecircRNAs are predominantly generated from back-spliced exons, where 3′ splice donors of the pre-mRNA are covalently linked to 5′ splice acceptors in reverse order [22]. Their formation mechanism mainly includes three models (Fig. 1).

**Fig 2:**
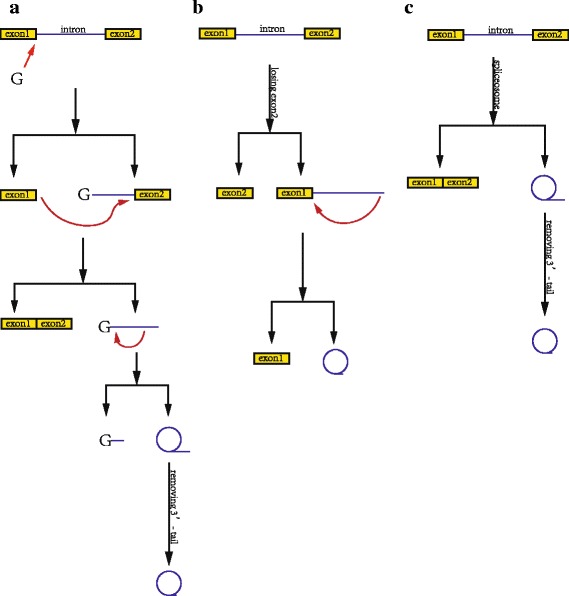
Biosynthesis of circular RNAs from introns. **a**. Circular RNA from group I introns. First, an exogenous guanosine(G) attacks the 5′-terminus of the intron as nucleophile. The 5′-exon is cut off due to the transesterification. Second, the 3′-hydroxyl of the free exon attacks the 5′-terminus of the 3′-exon as nucleophile, producing a linear intron. Third, a 2′-hydroxyl close to the 3′-terminus of the linear intron attacks a phosphodiester bond close to the 5′-terminus, producing an RNA lariat circularized with 2′,5′-phosphodiester and releasing the 5′-terminal sequence. Finally, the 3′- tail of the RNA lariat is removed. **b**. Circular RNA from group II introns. First, the RNA precursor releases the 3′-exon. Finally, the 2′-hydroxyl of the 3′-terminus attacks the 5′-terminus of the intron, producing an circular RNA circularized with 2′,5′-phosphodiester. **c**. Circular intron RNA(ciRNA). First, a pre-mRNA is spliced by a spliceosome, producing an RNA lariat circularized with 2′,5′-phosphodiester. Finally, the 3′- tail of the RNA lariat is removed
1.3. Exon-intron circRNAs are a type of circular RNA that is circularized with both exons and introns at the same time. Internal repeat sequences may play important roles in their generation, and they might be similar to ecircRNAs [5].
1.4. Recently, Yuan Gao et al. found intergenic circRNAs by CIRI (CircRNA Identifier), a novel circRNA identification tool that can detect circRNAs genome-wide. It contains two intronic circRNA fragments flanked by GT-AC splicing signals acting as the splice donor and acceptor of the circular junction while forming an integrated circRNA [24].

### Function of circular RNAs

#### Competing endogenous RNAs or miRNA sponges

Studies have proven that the circular RNAs CDR1as or ciRS-7 [11, 12], circ-SRY [12], circ-ITCH [8], circ- HIPK3 [13], hsa_circ_0000615 [14], and mm9_circ_012559 [15] are able to act as miRNA sponges. For example, the first observation of a circular RNA acting as a miRNA sponge is CDR1as, which has more than 70 conserved binding sites of miRNA-7 [25]. Moreover, circ-SRY is a circular RNA that is expressed specifically in mice testes and has 16 binding sites with miRNA-138 [12, 26].

Although the “miRNA sponge” is the classical model of circular RNA function, the applicability of the model is becoming more and more controversial. A recent study proved that most circular RNAs could not function as “bona fide” miRNA sponges [27].

### Interaction with RNA binding proteins

In addition to the function of miRNA sponges, circular RNAs can also interact with RNA binding proteins, such as circ-Foxo3 [10], circ-MBL (muscleblind) [9] and some ciRNAs [4]. Circ-Foxo3 is able to bind with many types of proteins. It can suppress the cell cycle and block the transition from G1 to S phase by interacting with CDK2 and p21 [10]. Additionally, researchers found that intron lariats can accumulate in the cytoplasm and bind with the protein TDP43, suppressing TDP43 toxicity in ALS (amyotrophic lateral sclerosis) [4].

### Modulating the stability of mRNAs

Some circular RNAs can modulate the stability of mRNAs. For example, circular antisense RNA from CDR1as can form a duplex structure with mRNA, stabilizing it [28]. Furthermore, in mouse macrophages, circ-RasGEF1B can strengthen the stability of mRNA of ICAM-1 [29].

### Regulating gene transcription

Circular RNAs can also regulate gene transcription. The mechanisms can be varied, such as combining with substance and sponging miRNAs. These circRNAs always exist in the nucleus. For example, both of the two exon-intron circular RNAs, circ-EIF3J and circ-PAIP2, can combine with the U1 snRNP to further interact with RNA Pol II and enhance the expression of their parental genes in HeLa and HEK293 cells [5]. As a result, EIciRNAs can play an important role in positive feedback regulation. CiRNAs can also regulate gene transcription. Researchers found that ci-ankrd52 and ci-sirt7 can also function as positive regulators of their parental gene transcription by interacting with Pol II [6]. This indicates that intron circular RNAs can also regulate parental gene transcription.

### Translating proteins

Recently, endogenous circRNAs were proven to be able to be translated into protein. Legnini I. et al. found that circ-ZNF609 could translate proteins in murine myoblasts when driven by IRES (internal ribosome entry site) [30]. Pamudurti N. R. et al. found circMbl3 could translate protein in fly heads [31]. Before this convincing evidence came out, most researchers believed that circRNAs were a distinct class of endogenous noncoding RNAs. However, some reports showed that a few circRNAs had the potential to be translated into proteins [32, 33]. The first circRNA found to be able to translate protein is the genome of the hepatitis δ virus-a single-stranded circular RNA, which could generate a protein of 122 amino acids [32]. Some reports showed that artificial circular RNAs with an IRES sequence upstream of the start codon can translate a functional GFP (green fluorescent protein) in vitro [34]. Other studies showed that synthesised circular RNAs with multiple FLAG-coding sequences could also translate proteins through a mechanism similar to rolling circle amplification (RCA) in the absence of any particular element for internal ribosome entry [33].

This discovery gives circRNAs a new function and provides a new direction for research of circRNA in the future. However, this discovery calls into doubt the notion of circRNAs being non-coding RNAs.

## Properties of CircRNAs in eukaryotic cells

### Stable structure

Circular RNAs from eukaryotic cells are stable in cells. Most exonic circular RNAs exhibit a half-life more than 48 h, while an average half-life for mRNAs is 10 h [35]. This may be due to their circular structure. Exonic circular RNAs and intronic circular RNAs are both resistant to RNase R [6, 36]. Therefore, they are potential stable molecular markers in diseases diagnosis and treatment.

### Incredible diversity and tissue-specific expression

More than 20,000 different circular RNAs from eukaryotes have been identified [37], and the number is expected to increase continuously. Many circular RNAs are expressed in specific tissues. For instance, circular RNAs from Rmst and Klhl2 are highly expressed in mouse brain, but not in liver or lung [16]. In Drosophila, circ-Mbl expression is much lower in the ovary than in the head [38].

### Abundance related with developmental stage or age

In different developmental stages, the expression of some circular RNAs can change. An experiment showed that the abundance of some circular RNAs changed with age. In the brain of a 22-month-old mouse, the expression of many circular RNAs such as mm9_circ_004501 and mm9_circ_013636 is increased compared to that of a 1-year-old mouse [39].

### Evolutionary conservation between different species

With technological breakthroughs in high-throughput deep sequencing, large numbers of circRNAs have been recently found in human [11, 20, 40], mouse [11, 20], nematodes [11, 41], zebrafish [42], *Drosophila* [43], protists [44] and plants [44]. Additionally, evolutionarily conserved sequences of circRNAs have been found. For example, it has been found that 457 human circular RNAs can map to murine genes, 69 of which contain homologous backsplice points [45].

## Circular RNAs as potential molecular markers of cancer

Recent studies suggest that circRNAs may play an important role in the initiation and development of cancer. It is possible that circRNAs can function as biomarkers of some cancers to support diagnosis. Present studies have partially proven that circRNAs could be molecular markers of tumours.

### CircRNAs in Gastric Cancer (GC)

Circ-PVT1 is found to be up-regulated in GC tissues via the amplification of its genomic locus and it may promote GC cell proliferation by acting as a sponge against members of the miR-125 family [19]. Recently, hsa_circ_0000190 was found to be a potential biomarker for the diagnosis of gastric cancer. It was down-regulated in both GC tissues and plasma from patients with GC. Compared with CEA (carcinoembryonic antigen) and CA19-9, two classic biomarkers for GC, hsa_circ_0000190 has better sensitivity and specificity [46].

For some tumours of digestive tract, some classic biomarkers may not be specific enough. For example, CEA from plasma can be up-regulated in GC, pancreatic and colon cancers. Thus, auxiliary diagnosis using biomarkers generally needs more than one biomarker. However, tissue-specific expression of circular RNAs may help solve the problem of specificity.

### CircRNAs in Hepatocellular Carcinoma (HCC)

Hepatocellular carcinoma is the third most common cause of cancer deaths worldwide [47]. The prognosis is still poor because of its aggressiveness and recurrence rate. Thus, efficient biomarkers are necessary for better early diagnosis and prognosis analysis. To date, many biomarkers have been proposed in the diagnosis and prognosis of HCC, such as alpha-fetoprotein (AFP), Lens culinaris-agglutinin-reactive fraction of AFP (AFP-L3), protein induced by vitamin K absence orantagonist-II (PIVKA-II), vascular endothelial growth factor (VEGF), hypoxia-inducible factor (Hif), and so on [48]. AFP is the most efficient biomarker for HCC diagnosis, but a study has shown that in up to 40% of patients with HCC, AFP expression is at a normal level, which reflects a low sensitivity [49]. The combination of AFP, AFP-L3, PIVKA-II would have a better sensitivity and specificity in the diagnosis of HCC. Additionally, in HCC tissue, high expression of VEGF and Hif are related to early recurrence after resection, which reflects significance as a prognostic biomarker.

On the other hand, there is increasing evidence that circRNAs are associated with the development and invasion of HCC, despite their still unclear role [50]. Xingchen Shang et al. found that there is a significant difference in the expression of hsa_circ_0005075 between HCC and normal hepatocellular tissues (P < 0.001) and that its expression correlates with HCC tumour size (P = 0.042) [50]. Similarly, Meilin Qin et al. found a dramatic discrepancy in the expression of hsa_circ_0001649 in HCC cells and adjacent liver tissues, specifically a down-regulation in HCC [51], reflecting its potential as a diagnosis and prognosis biomarker.

However, what is the relation between circular RNAs and traditional biomarkers? Recent research showed that the high expression of ciRS-7 in HCC is related to hepatic MVI (microvascular invasion) and AFP levels [52]. Furthermore, hsa_circ_0001649 has potential binding sites for miR-182 [51] and hsa_circ_0005075 also has potential binding sites for miR-93 [50]. miR-182 and miR-93 can respectively function as potential prognosis and diagnosis biomarkers for HCC [48].

From our point of view, we think that circRNAs can serve as potential diagnostic and prognostic biomarkers for HCC. Their expression is correlated to some traditional biomarkers, such as AFP and some miRNAs. They might interact with miRNAs, serving as miRNA sponges to regulate downstream target gene expression and influencing the development of HCC. Furthermore, compared with miRNAs, circRNAs are more stable and can even be detected in exosomes. Thus, we believe that the combined use of circRNAs and traditional biomarkers may provide better clinical utility for HCC.

### CircRNAs in lung cancer

Lung cancer, which causes cancer-related deaths worldwide, comprises non-small-cell lung cancer (NSCLC) and small-cell lung cancer. Statistical analysis shows that NSCLC accounts for 85% of all lung cancers [53]. To help predict the occurrence and development of NSCLC, doctors use biomarkers in the clinic. Biomarkers used for predicting include the anaplastic lymphoma kinase (ALK) fusion oncogene and sensitising epidermal growth factor receptor (EGFR) mutations. Other biomarkers include HER2, BRAF, NUT, MET, ROS1, DDR2, FGFR1, KRAS, and PTEN with point mutations and rearrangements in the gene [54]. However, these traditional biomarkers have the disadvantage of relatively lower positive detection rates and lower organ specificity. Nonetheless, with the development of RNA-seq and bioinformatics, some researchers found microRNAs that are correlated with lung cancer. Cheng Wang et al. have demonstrated that miR-483-5p, miR-193a-3p, miR-25, miR-214 and miR-7 can potentially be used as biomarkers for diagnosing NSCLC patients [55].

Compared with these traditional biomarkers, circRNAs may compensate for low organ specificity because of its diversity and tissue-specific expression. Li Wan et al. found that circ-ITCH is overexpressed in lung cancer tissue and can inhibit the activation of the Wnt/β-catenin signalling pathway by acting as a sponge of miR-7 and miR-214 [56], competing with these miRNAs for binding sites of ITCH. The Wnt/β-catenin pathway plays an important role in tumour initiation, progression, and metastasis if some of its factors are aberrantly activated [57]. Wnt/β-catenin signalling is inhibited by the E3 ubiquitin protein ligase ITCH via ubiquitination and degradation of phosphorylated Dishevelled 2 [58]. Wnt/β-catenin was inhibited by circ-ITCH in oesophageal squamous cell carcinoma (ESCC) to regulate the cell cycle [8] and in colorectal cancer [59] (CRC).

Coincidentally, up-regulated circRNA 100876 in NSCLC was found to closely correlate with lymph node metastasis (P = 0.001) and tumour staging (P = 0.001). This indicated that circRNA 100876 could be a potential prognostic biomarker for NSCLC [60].

Consequently, circRNAs can supplement traditional biomarkers of NSCLC to increase the positive detection rate. Compared with other non-coding RNAs that show potential to serve as the biomarkers of cancer, circRNAs have higher stability, high abundance, and high conservation as previously mentioned. CircRNAs may thus be more suitable biomarkers.

### CircRNA in colon carcinoma

CEA is used to monitor relapse of colorectal cancer, and checking KRAS gene mutation can assist in diagnosing colorectal cancer. However, CEA always has positive expression in intermediate and terminal cancer and has nothing to do with early diagnosis and differential diagnosis of colorectal cancer [61]. Yongchao Dou et al. used three isogenic colorectal cancer cell lines that differ only in KRAS mutation status including DLD-1 (containing both wild-type and G13D mutant KRAS [KRAS proto-oncogene, GTPase] alleles and isogenically-matched derivative cell lines), DKO-1 (mutant KRAS allele only), and DKs-8 (wild-type KRAS allele only), and found that most circRNAs are down-regulated in KRAS mutated colorectal cancer cell lines. Number is 443 and 305 in DKO-1 and DLD-1 cells, respectively (False Discovery Rate [FDR] < 0.01 and Fold Change [FC] > 2). In contrast, only 5 and 13 circRNAs were significantly up-regulated in DKO-1 and DLD-1 cells, respectively [62]. Furthermore, in all three cell lines, circRNAs were more abundant in exosomes than in cells [62]. This evidence indicates that circRNAs correlate with mutations of KRAS. CircRNAs serving as promising colorectal cancer biomarkers may improve the positive detection rate of KRAS, which assists in predicting or diagnosing colorectal cancer.

### Fusion-CircRNAs in leukaemia

Promyelocytic leukaemia-retinoic acid receptor α (PML-RARα) is a fusion protein that plays an important role in acute promyelocytic leukaemia (APL). PML-RARα can be cleaved by neutrophil elastase and generate a mutational product, PML (NLS-), which can act as a novel diagnostic biomarker for APL and had a sensitivity and specificity of 92.6 and 77.3%, respectively [63].

Recent studies have also shown that in leukaemia with PML/RARα translocations, a type of special fusion-circRNA can be generated during the generation of fusion-gene [64]. We thus considered if these fusion-circRNAs might associate with PML(NLS-) and serve as potential combined biomarkers. Furthermore, the study showed that aside from leukaemia, fusion-circRNAs have been found to be expressed in SK-HEP-1 osteosarcoma cell lines and H3112 lung carcinoma cell lines, suggesting that they might be used as pathognomonic markers for other tumour types. In vitro and in vivo, fusion-circRNAs are also able to increase cellular proliferation rates and make contributions to cellular transformation and tumorigenesis, indicating that they can also serve as a potential prognosis biomarker and treatment target.

Thus, this evidence suggests that fusion-circRNAs, as products of the PML-RARα fusion gene, are very likely to be a diagnostic biomarker or therapeutic target in leukaemia. Combining the fusion-circRNAs and other products of the PML-RARα fusion gene such as PML(NLS-) might give a better diagnosis efficiency for leukaemia.

Additionally, there were significant differences between tumour and normal tissues in laryngeal squamous cell cancer (LSCC) [65], bladder carcinoma [66], and ovarian tumours [67], which provide the possibility for circRNAs as potential biomarkers.

## Conclusions

Circular RNAs are a new class of non-coding RNAs attracting more and more researchers’ attention. They may play an important role in gene expression and signalling pathways and participate in the development of some diseases. As mentioned above, circular RNAs have shown tremendous potential for diagnosing tumours. Circular RNAs can exist in exosomes and blood plasma because of their stability. Studies shows that ecircRNAs in exosomes were enriched by at least 2-fold compared to those in its producer, MHCC-LM3 liver cancer cells and the ratio of circRNA to linear RNA was approximately 6-fold higher than that in cells [68]. In colorectal cancer patients, 67 circRNAs were lost and 257 new circRNAs were found compared to healthy subjects, and the expression levels of circ-KLDHC10 were significantly increased in cancer serum compared to normal serum [68]. On the other hand, in plasma samples of gastric cancer patients, hsa_circ_0000190 was found to be significantly down-regulated, and the expression level of hsa_circ_002059 in postoperative gastric cancer patients was found to be significantly different from that of preoperative gastric cancer patients. Thus, compared with tumour tissues, the stable existence of circular RNA in exosomes and plasma provide a more convenient way for diagnosing cancer. At present, molecular biomarkers used in the clinic are always proteins that have low organ specificity. For example, CEA is common in gastrointestinal tumours, and CA19-9 is common in varied glandular cancer. If circRNAs as biomarkers of cancers are to be used in the clinic, their specific expression may assist in solving the problem of existing markers’ low organ specificity.

However, circRNAs also have some disadvantages for diagnosis. First, some circRNAs need patient tissue for diagnosis, which causes some trauma for patients. Second, detecting circRNAs in tissue or in exosomes is more expensive than existing checks, which limits the widespread use of circRNAs as a biomarker. Third, the reliability of using circRNAs for diagnosis still needs to be proven. Furthermore, due to the enormous number of circular RNAs, their functions may be complex and different from each other. Current studies mainly focus on the interactions between RNAs and proteins, but the function of their secondary structures and their roles in DNA genome are still unknown. Can endogenous circular RNAs in eukaryotic cells be translated naturally? How are circular RNAs degraded in the cell? Although the generation of circular RNAs and their definite functions are still not totally clear, with the development of related studies, there is no doubt that more and more important roles in disease prevention, diagnosis and treatment will be found in the future.

## Abbreviations

AFP: Alpha fetal protein
AFP-L3: Lens culinaris-agglutinin-reactive fraction of AFP
APL: Acute promyelocytic leukemia
CEA: Carcinoembryonic antigen
circRNAs: Circular RNAs
CIRI: CircRNA Identifier
ciRNAs: Circular intronic RNAs
CRC: Colorectal cancer
ecircRNA: Exonic circRNA
EIciRNA: Exon-intron circRNAs
ESCC: Esophageal squamous cell
GC: Gastric cancer
HCC: Hepatocellular carcinoma
Hif: Hypoxia-inducible factor
IRES: Internal ribosome entry site
LSCC: Laryngeal squamous cell cancer
NSCLC: Non-small-cell lung cancer
RCA: Rolling circle amplification
RNA-seq: RNA sequencing
snRNP: Small nuclear ribonucleo proteins
